# Silibinin Therapy Improves Cholangiocarcinoma Outcomes by Regulating ERK/Mitochondrial Pathway

**DOI:** 10.3389/fphar.2022.847905

**Published:** 2022-03-23

**Authors:** Yang Bai, Jiaqi Chen, Weijian Hu, Lei Wang, Yulian Wu, Shi’an Yu

**Affiliations:** ^1^ Department of Surgery, The Second Affiliated Hospital, Zhejiang University School of Medicine, Hangzhou, China; ^2^ Department of Hepatobiliary and Pancreatic Surgery, Affiliated Jinhua Hospital, Zhejiang University School of Medicine, Jinhua, China; ^3^ Stomatology Hospital, School of Stomatology, Zhejiang University School of Medicine, Clinical Research Center for Oral Diseases of Zhejiang Province, Key Laboratory of Oral Biomedical Research of Zhejiang Province, Cancer Center of Zhejiang University, Hangzhou, China; ^4^ Department of Urology Surgery, Affiliated Jinhua Hospital, Zhejiang University School of Medicine, Jinhua, China

**Keywords:** silibinin, cholangiocarcinoma, ERK, mitochondrial membrane potential, anti tumor drug

## Abstract

**Background:** Silibinin is widely utilized drug in various cancer treatments, though its application in cholangiocarcinoma has not yet been explored. For the first time, we evaluated the anticancer potential and underlying molecular mechanism of silibinin in treatment of cholangiocarcinoma treatment.

**Methods:** HuCCT-1 and CCLP-1 cells were chosen to be an *in vitro* study model and were exposed to various concentrations of silibinin for indicated times. Cell viability was evaluated by the cell counting kit-8 (CCK-8) assay and half maximal inhibitory (IC50) concentrations were calculated. Cell proliferation capacity was determined through the use of colony formation and 5-Ethynyl-2′- deoxyuridine (EdU) assays. Cell apoptosis and cycle arrest were assessed by Live/Dead staining assay and flow cytometry (FCM). The protein levels of extracellular regulated protein kinases (ERK)/mitochondrial apoptotic pathway were evaluated through western blotting (WB). Mitochondrial membrane potential changes were determined *via* 5,5′,6,6′-Tetrachloro-1,1′,3,3′-tetraethyl-imidacarbocyanine iodide (JC-1). A cholangiocarcinoma cell line xenograft model was used to assess the anti-tumor activity of silibinin *in vivo*.

**Results:** Inhibition of the ERK protein by silibinin led to a significant decrease in mitochondrial membrane potential, which, in turn, caused Cytochrome C to be released from the mitochondria. The activation of downstream apoptotic pathways led to apoptosis of cholangiocarcinoma cells. In general, silibinin inhibited the growth of cholangiocarcinoma cell line xenograft tumors.

**Conclusions:** Silibinin is able to inhibit cholangiocarcinoma through the ERK/mitochondrial apoptotic pathway, which makes silibinin a potential anti-tumor drug candidate for cholangiocarcinoma treatment.

## Introduction

Cholangiocarcinoma is a highly malignant tumor that originates from the bile duct epithelial cells and occurs from the capillary bile duct to the common bile duct. It is the second most common hepatobiliary pancreatic tumor in the world, accounting for 3% of all gastrointestinal malignancies (Banales et al., 2020). Cholangiocarcinoma exhibits different biological behavior than hepatocellular carcinoma, as cholangiocarcinoma is more likely to infiltrate into the bile duct wall, and invade the surrounding liver tissue, blood vessels, nerves and lymphoid tissue. Therefore, the surgical resection rate is low and the overall prognosis of patients remains poor (Rizvi et al., 2018). Thus far, most patients are in the advanced stage of the disease at time of diagnosis, and systemic treatment remains the main treatment (Kelley et al., 2020). However, due to differences in molecular pathology and gene mutations between biliary systemic tumors at different anatomical sites, the effects of systematic treatments are quite different (Chaisaingmongkol et al., 2017; Montal et al., 2020). Therefore, there is an urgent need to develop novel drugs for the treatment of cholangiocarcinoma.

Silibinin is an antioxidant that has been extracted from a plant called milk thistle (Bosch-Barrera et al., 2017). It can stabilize the hepatocyte membrane and maintain its integrity. It is also able to promote the recovery of hepatocyte ultrastructure, as it is the most effective flavonoid found in the world (Salomone et al., 2017). Additionally, studies have demonstrated that silibinin can also inhibit growth and differentiation of liver cancer (Mao et al., 2018), prostate cancer (Vue et al., 2016), breast cancer (Si et al., 2020) and cervical cancer (García-Maceira and Mateo, 2009). It is very well-known that the malignant degree of cholangiocarcinoma is high, and conventional treatment is limited. Therefore, in this study, we evaluated the use of silibinin for the treatment of cholangiocarcinoma in order to find a novel treatment for this disease.

ERK is a member of the mitogen activated protein kinase (MAPK) family (Guo et al., 2020). After it becomes activated, it has an important role in cell growth, development, division, death, as well as malignant transformation. ERK-mediated signal transduction pathway receives various mitogen stimulation, as well as stress of cells from inside and outside. It activates ERK substrate, and regulates cell proliferation, apoptosis and invasion (Lavoie et al., 2020). Activated ERK plays an anti-apoptotic role by phosphorylating the anti-apoptotic molecule Bcl-2, activating transcription factors and interfering with TRAIL, which finally promotes tumor cell growth. Overactivation of ERK has been found in oral cancer (Gao et al., 2020), melanoma (Savoia et al., 2019), breast cancer (Peng et al., 2017) and others. However, there are few studies on ERK protein in cholangiocarcinoma. In this paper, we were aiming to observe the therapeutic effect of silibinin on cholangiocarcinoma *in vivo* and *in vitro*, as well as to explore its potential mechanism.

## Material and Methods

### Materials

Silibinin (cat. no. S109809) was purchased from Aladdin. Primary antibodies against Caspase-9 (cat. no. ab202068), Caspase-3 (cat. no. ab184787), PARP (cat. no. ab191217), p53 (cat. no. ab26) and GAPDH (cat. no. ab181602) were bought from Abcam. CDK2 (cat. no. 10122-1-AP), cdc25a (cat. no. 55031-1-AP), ERK (cat. no. 16443-1-AP), CDK4 (cat. no. 11026-1-AP), Bcl-2 (cat. no. 12789-1-AP), Bcl-xl (cat. no. 10783-1-AP), and Cytochrome C (cat. no. 10993-1-AP) were purchased from Proteintech. Cyclin E2 (cat. no. #4132) was bought from Cell Signaling Technology. p-ERK (cat. no. AF1015) and VDAC (cat. no. DF6140) were bought from Affinity. The antibodies were utilized at the recommended concentrations following the manufacturer’s instructions.

### Cell Culture

HuCCT-1 and CCLP-1, two human cholangiocarcinoma cell lines, were acquired from the American Tissue Culture Collection (Manassas, VA, USA). The cell lines were cultured in RPMI 1640 medium (cat. no. 01-100-1A, Biological Industries) that was supplemented with 10% FBS (cat. no. 04-001-1B, Biological Industries), 100 U/ml penicillin and 100 μg/ml streptomycin (Invitrogen, CA, USA). All cells were incubated at 37°C in a humidified atmosphere with 5% CO_2_.

### Cell Counting Kit-8 Assay

For concentration-dependent cell viability experiments, cells were seeded onto 96-well plates (4*10^3^ cells/well), 18 h prior to treatment with varying concentrations of silibinin for an additional 48 h. Overall, 10 μl CCK-8 (cat. no. C0037, Beyotime) was added into each well for 2 h, and the absorption value was then measured. For time-dependent cell viability experiments, HuCCT-1 and CCLP-1 cells were plated onto 96-well plates 18 h prior to treatment with an IC50 and 2*IC50 concentrations of silibinin for 0, 6, 12, 24, 48, and 72 h. The absorption value was measured at different time points. Curves were fitted and analyzed using the GraphPad Prism 8.4.0 software (GraphPad Software, Inc.).

### Colony Formation Assay

Cholangiocarcinoma cells were seeded onto a 6-well plate (Jet Bio-Filtration Co., Ltd.) at 5,000 cells per well 18 h prior to treatment with silibinin or SCH772984. Then, after treatment cells were incubated for another 14 days and then stained with crystal violet. More than 50 cells were defined as a clone.

### EdU Proliferation Assay

The BeyoClick™ EdU Cell Proliferation Kit with Alexa Fluor 594 (cat. no. C0078S; Beyotime) was used to evaluate the effect of silibinin and SCH772984 on the cholangiocarcinoma cell lines, according to the manufacturer’s instructions. After treatment for 48 h, cholangiocarcinoma cells were EdU-labeled and cultured for another 2 hours. Next, Hoechst33342 staining was carried out. Fluorescence microscopy (Olympus Corporation, Japan) was utilized to take a picture.

### Apoptosis and Cell Cycle Analysis

Cell apoptosis was assessed using Annexin V-FITC Apoptosis Detection Kit (cat. no. C1062M, Beyotime), according to the manufacturer’s protocol. In brief, cells were seeded onto six-well plates at a density of 4*10^5^ cells per well. After being exposed to silibinin or SCH772984 for 48 h, cells were then cultured with fluorescein isothiocyanate isomer (FITC) and propidium iodine (PI) at 37°C for 30 min. Then, cell cycle distribution was evaluated utilizing the Cell Cycle and Apoptosis Analysis Kit (cat. no. C1052, Beyotime). After exposure to IC50 and 2*IC50 concentrations of silibinin for 48 h, the cells were collected and fixed with 75% pre-cooled ethanol. Then, cells were stained with PI for 30 min prior to detection. Finally, apoptosis and cell cycle distribution were detected by FCM, and then analyzed utilizing Flowjo 10.4 (FlowJo LLC) and Modifit LT 5 (Verity Software House, Inc.).

### Live/Dead Staining Assay

Live/dead staining assay was conducted utilizing a Calcein/PI Live/Dead Viability/Cytotoxicity Assay Kit (cat. no. C2015M, Beyotime). In brief, after exposure to IC50 and 2*IC50 concentrations of silibinin for 48 h, cells were collected and stained using Calcein and PI for 20 min at room temperature. The pictures were taken by a fluorescence microscope (Olympus IX71).

### Cell Mitochondria Isolation

Mitochondria isolation assay was carried out according to the manufacturer’s protocol. Briefly, after exposure to IC50 and 2*IC50 concentrations of silibinin for 48 h, cells were collected and then incubated with mitochondria isolation reagents that contain 1% Phenylmethanesulfonyl fluoride (PMSF) in an ice bath for 15 min. Subsequently, cell suspensions were then homogenized for appropriate times, and the proportion of cells that were positive for trypan blue staining was examined microscopically (Supplementary Figure S1A). When the positive ratio exceeded 50%, then the homogenate was stopped and mitochondria were isolated using differential centrifugation.

### Western Blotting

Western blotting analysis was carried out, as previously described (Chen et al., 2021). Briefly, cells were collected and dissolved within the Lysis Buffer (cat. no. P0013B, Beyotime) containing 1 mM PMSF (cat. no. ST506, Beyotime). The protein concentration was detected through the use of an enhanced BCA Protein Assay Kit (cat. no. P0010, Beyotime). Next, equivalent amounts of protein were separated on SurePAGE Bis-Tris gels (GenScript) for electrophoresis, and then transferred to polyvinylidene fluoride (PVDF) membranes. Then, the membranes were blocked *via* a blocking buffer (cat. no. P0252, Beyotime) for 1 h, and then cultured with primary antibodies overnight. After incubation with secondary antibodies at appropriate concentration for an hour at room temperature, the protein bands on the membrane were eventually identified using a Hypersensitivity Chemiluminescence Kit (cat. no. G2020, Servicebio). The band intensity was quantified using the ImageJ software (National Institutes of Health).

### Mitochondrial Membrane Potential Analysis

Briefly, after exposure to IC50 and 2*IC50 concentrations of silibinin for 48 h, cells were collected and then incubated with the JC-1 staining working solution at 37°C for 30 min. The treated cells underwent subsequent FCM or fluorescence photographs by confocal microscopy.

### *In Vivo* Experiments

Animal experiments were carried out, as described previously (Chen et al., 2021). Until tumors reached approximately 30 mm^3^, the mice were randomly assigned into three different groups. First, the mice were assigned to a control group (*n* = 4; 0.9% physiological saline). Second, the mice were assigned to a low-dose silibinin group (*n* = 4; 100 mg/kg). Third, they were assigned to a high-dose silibinin group (*n* = 4, 200 mg/kg). Finally, compounds were administered in a peritumoral manner once a day. Tumor volume was calculated using the following formula: volume = length*width^2^/2 and mice were sacrificed 3 weeks after injection.

### Immunohistochemistry Staining Analysis

For immunohistochemistry (IHC) staining assay, sections that were 5 um thick were stained with hematoxylin and eosin (HE) for analysis of proliferation and apoptosis. For total ERK (t-ERK) and phosphorylated ERK (p-ERK) detection of protein expression, tissue sections were incubated with anti-ERK and p-ERK primary antibodies overnight, and then incubated with HRP-conjugated secondary antibody. The proliferative capacity of tumors was analyzed using Ki-67 staining, and apoptotic cells were determined through the use of a TdT mediated dUTP Nick End Labeling (TUNEL) Apoptosis Assay Kit (cat. no. C1091, Beyotime), as per the manufacturer’s introductions.

### Statistical Analysis

Data and figures were processed through the use of GraphPad Prism 8.4.0 (GraphPad Software, Inc.). The values were expressed as mean ± standard deviation, and then assessed using Student’s t-test or the one-way analysis of variance procedure. Dunnett’s multiple comparisons test was chosen as the post test for data satisfying the homogeneity of variance. Furthermore, Dunnett’s T3 multiple comparisons test was chosen as a post-hoc test for data that did not meet the homogeneity of variance. With the exception of animal experiments (four mice allocated per group to ensure a sufficient group size for statistical analysis), which was carried out once, all other experiments were performed in triplicate with at least three independent experiments. *p < 0.05* was considered to be a statistically significant difference.

## Results

### The Viability of Cholangiocarcinoma Cells was Significantly Inhibited by Silibinin

**FIGURE 1:**
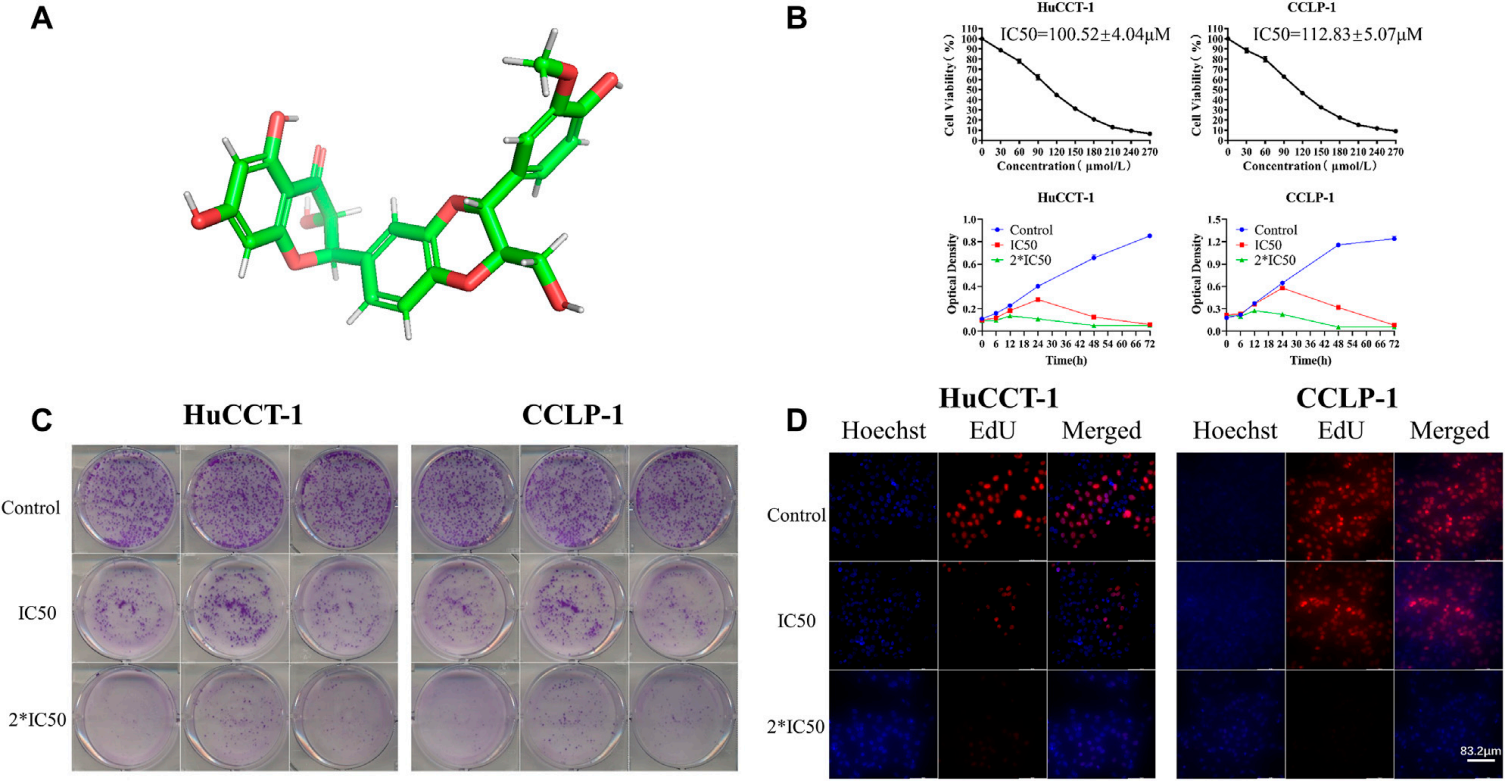
Silibinin inhibits cell viability in cholangiocarcinoma cells. **(A)**. Diagram of the three-dimensional structure of silibinin. **(B)**. Silibinin inhibited cell viability of HuCCT-1 and CCLP-1 cell lines in a concentration-dependent and time-dependent manner. Cells were treated with varying concentrations of silibinin for indicated time points (6, 12, 24, 48 and 72 h) and cell viability was examined by the CCK-8 assay. **(C)**. Colony formation assay results of HuCCT-1 and CCLP-1 cell lines treated with different concentrations of silibinin. **(D)**. EdU proliferation assay results of HuCCT-1 and CCLP-1 cell lines treated with different concentrations of silibinin.

In order to illustrate the therapeutic efficacy of silibinin (Figure 1A)on cholangiocarcinoma, two cholangiocarcinoma cell lines, HuCCT-1 and CCLP-1, were chosen to be *in vitro* cell models. The cell viability of HuCCT-1 and CCLP-1 was significantly inhibited by silibinin after administering the drug for 48 h and the IC50 was 100.52 ± 4.04 μmol/L and 112.83 ± 5.07 μmol/L, respectively (Figure 1B). In addition, cholangiocarcinoma cells were inhibited by silibinin in a time-dependent manner. The ability to proliferate is one aspect of cell viability. Therefore, the proliferation of cholangiocarcinoma after silibinin treatment was determined. The colony formation ability and the proliferation ability of cholangiocarcinoma cells treated with silibinin were significantly inhibited (Figures 1C,D). These results illustrate that silibinin can act as a tumor suppressor by inhibiting the proliferation of cholangiocarcinoma.

### Apoptosis was Induced by Silibinin in Cholangiocarcinoma Cells

**FIGURE 2:**
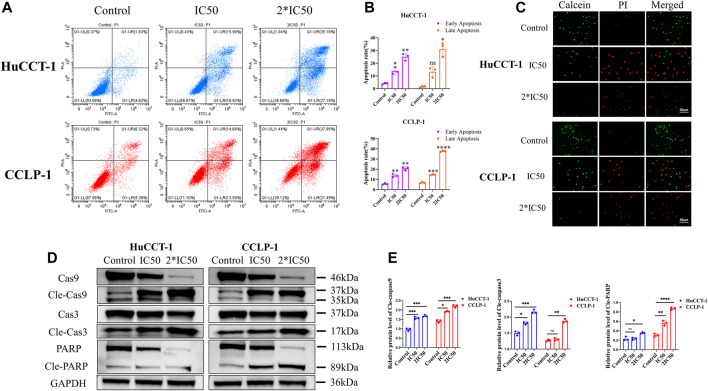
Silibinin induced apoptosis in HuCCT-1 and CCLP-1 cells. **(A,B)**. Detection of apoptosis by FCM and quantitative analysis of the apoptosis rate. HuCCT-1 and CCLP-1 cells were treated with IC50 and 2*IC50 concentrations of silibinin for 48 h **(C)**. The live/dead staining assay results of the HuCCT-1 and CCLP-1 cell lines treated with silibinin for 48 h. Red dots represent dead cells and green dots represent surviving cells. **(D)**. Western blotting results of HuCCT-1 and CCLP-1 cell lines treated with IC50 and 2*IC50 concentrations of silibinin for 48 h. The apoptosis-related proteins were identified. **(E)**. The quantification results of cle-caspase-9, cle-caspase-3 and cle-PARP. Data are shown as mean ± SD. *p < 0.05, **p < 0.01, ***p < 0.001, ****p < 0.0001, significantly difference compared to the control group.

In addition to their proliferative capacity, apoptosis can significantly affect cell viability as well. Therefore, the effect of silibinin on cholangiocarcinoma cell apoptosis was examined. After treatment with IC50 and 2*IC50 concentrations of silibinin for 48 h, the apoptosis rate of HuCCT-1 and CCLP-1 cells was significantly increased compared to the control group, as shown by FCM with FITC/PI double staining (Figures 2A,B). Consistent with results from FCM, Live/Dead cell staining assay indicated a significant transition from calcein signal to PI signal (Figure 2C), demonstrating that silibinin induced apoptosis of cholangiocarcinoma cells in a dose-dependent manner. In order to further determine whether cholangiocarcinoma cells underwent apoptosis, rather than necrosis or other types of cell death, western blotting was utilized to determine the expression of the downstream apoptosis-related proteins, Caspase-3 and PARP. The expression of cleaved Caspase-3 and cleaved PARP1 significantly increased after silibinin treatment, which means that silibinin induced cholangiocarcinoma cell death by activating apoptosis pathways (Figures 2D,E). Furthermore, we discovered that expression of cleaved Caspase-9 significantly increased after silibinin administration, which implies that apoptosis of cholangiocarcinoma cells may be mitochondria-related.

### G1 Arrest was Induced by Silibinin in Cholangiocarcinoma Cells

**FIGURE 3:**
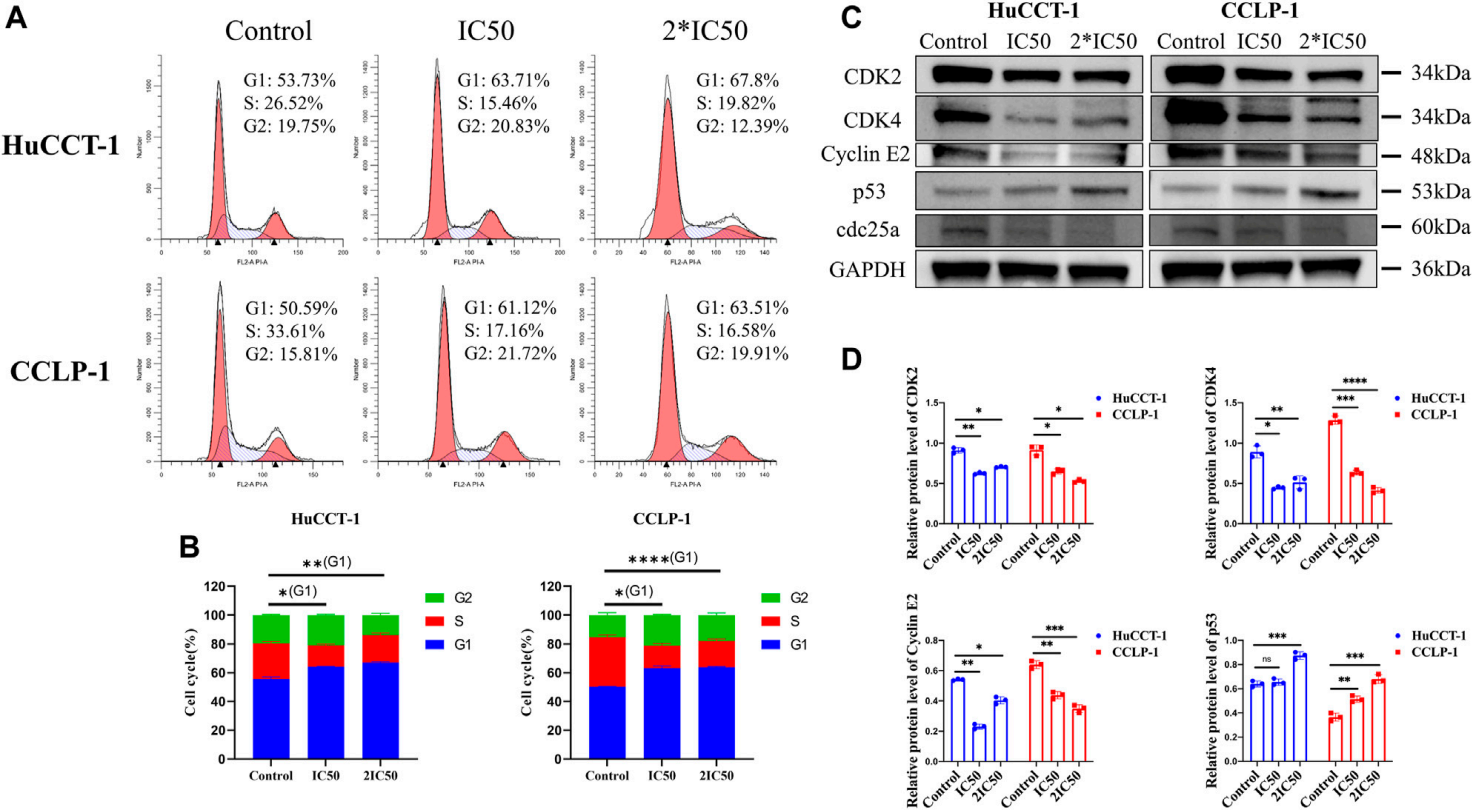
Silibinin induced G1 arrest in cholangiocarcinoma. **(A,B)**. Cholangiocarcinoma cell cycle distribution was determined using FCM analysis after treatment with IC50 and 2*IC50 concentrations of silibinin. The histograms show the percentage of HuCCT-1 and CCLP-1 cells in each phase. **(C,D)**. Western blotting results of G1 checkpoint proteins. Cells were treated with IC50 and 2*IC50 concentrations of silibinin for 48 h. The quantification results of CDK2, CDK4, Cyclin E2 and p53 are demonstrated by histograms. Data are shown as mean ± SD, *p < 0.05, **p < 0.01, ***p < 0.001, ****p < 0.0001, significantly difference compared with control group.

In order to explore more underlying mechanisms resulting in loss of cell viability after silibinin treatment, FCM was utilized to evaluate the cell cycle distribution of both cell lines. The percentage of cells in the G1 phase increased significantly in the HuCCT-1 and CCLP-1 cell line after being treated with IC50 concentration of silibinin for 48 h, from 53.73 to 63.71% (*p < 0.05*) and from 50.59 to 61.12% (*p < 0.05*), respectively (Figures 3A,B). This ratio was further enhanced when 2*IC50 concentration of silibinin was administered. Next, the expression of the G1 checkpoint protein was determined by WB. The CDK2, CDK4 and Cyclin E2 levels decreased markedly with a significant increase in p53, indicating that treatment with silibinin might result in G1 arrest in cholangiocarcinoma cells (Figures 3C,D).

### Silibinin-Mediated Apoptosis of Cholangiocarcinoma Cells was Associated with Mitochondrial Apoptosis

**FIGURE 4:**
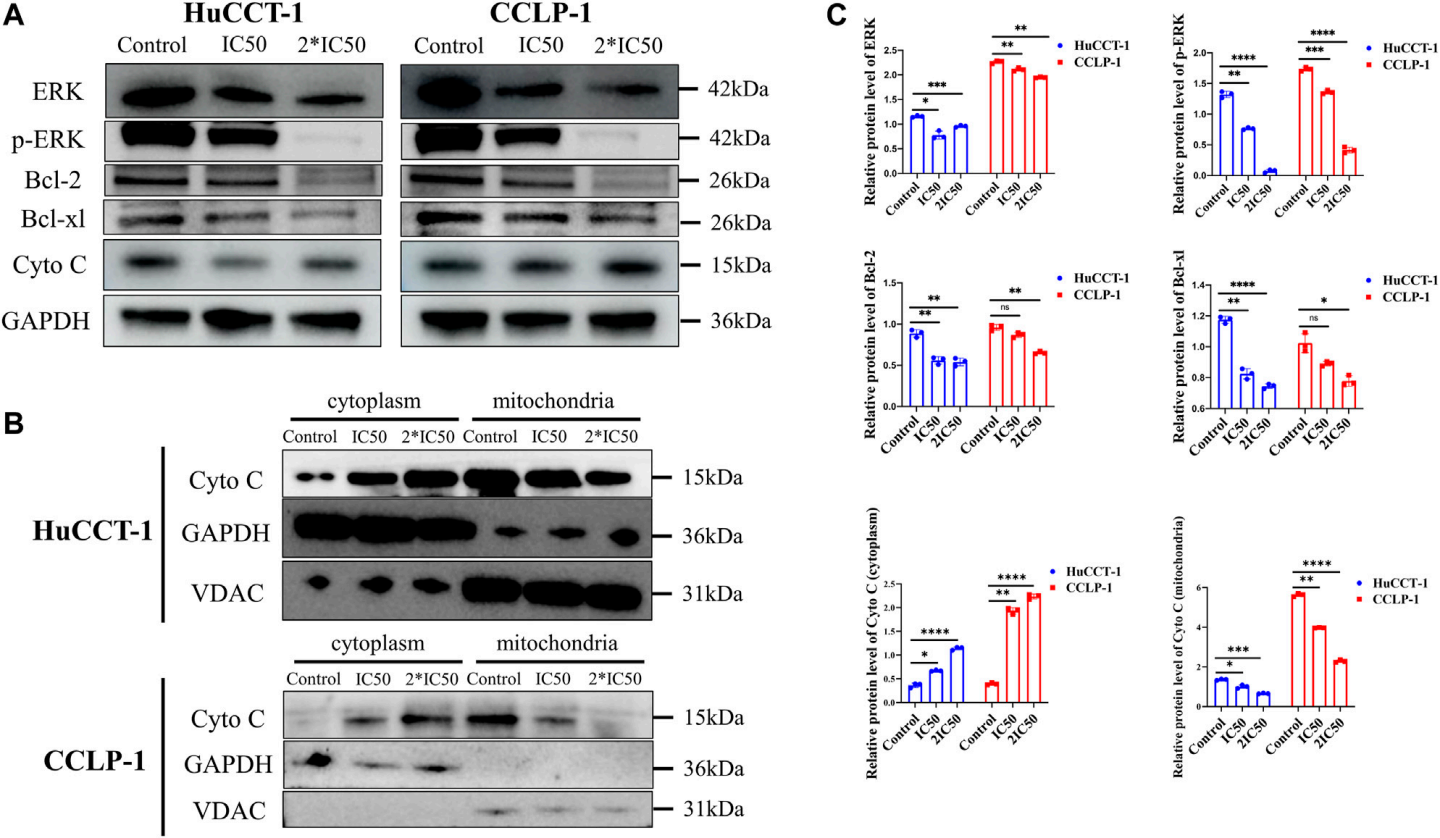
Silibinin induced apoptosis of cholangiocarcinoma cells through the ERK/Cytochrome c pathway. **(A)**. Expression of upstream proteins of the mitochondrial apoptosis pathway, as detected by western blotting. Cells were treated with IC50 and 2*IC50 concentrations of silibinin for 48 h **(B)**. Cytochrome C expression in different parts after mitochondrial isolation. **(C)**. The quantification results of western blotting. Data are shown as mean ± SD, *p < 0.05, **p < 0.01, ***p < 0.001, ****p < 0.0001, significantly different compared to the control group.

**FIGURE 5:**
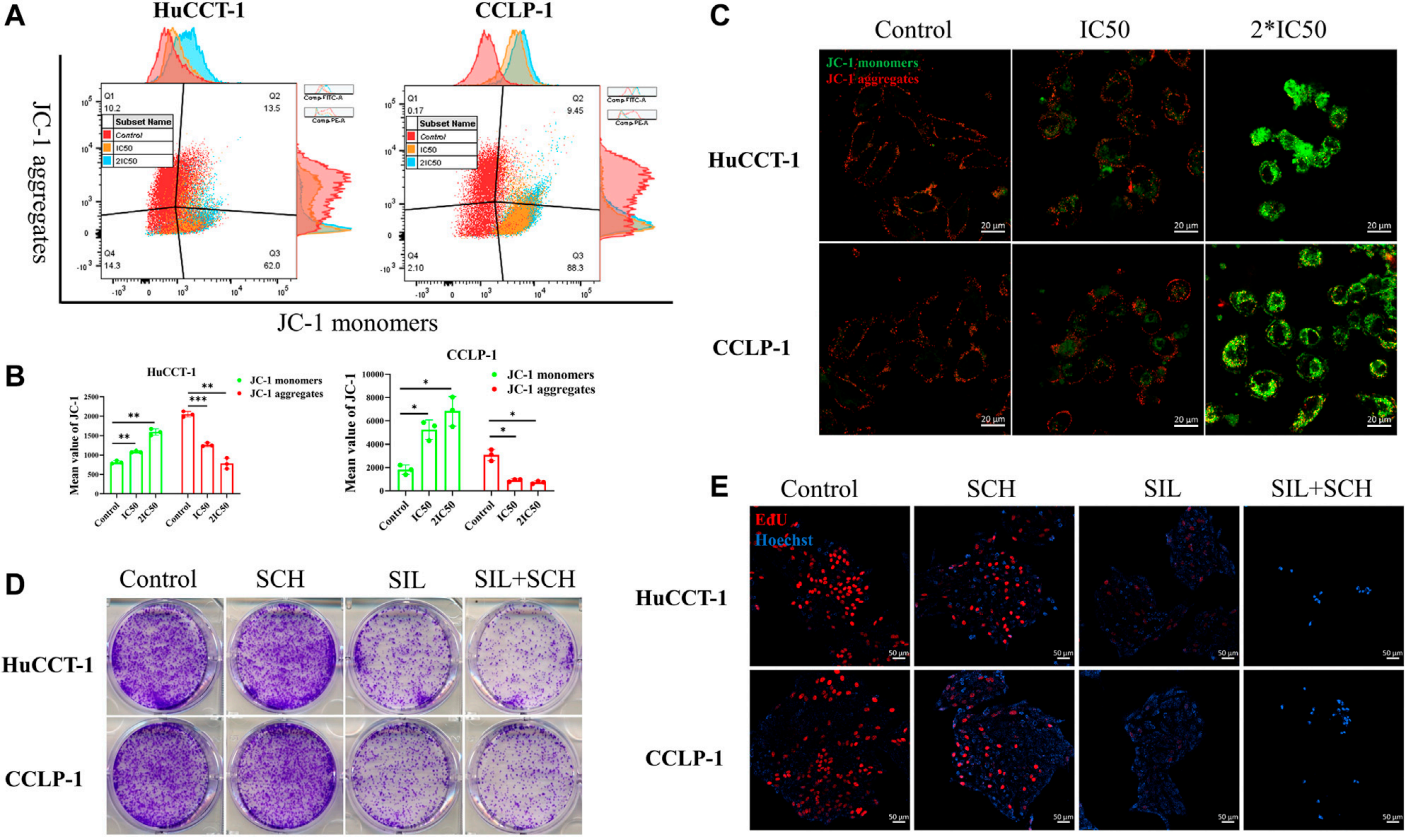
Silibinin acts on the ERK protein, causing a decrease in mitochondrial membrane potential. **(A,B)**. FCM results of the mitochondrial membrane potential changes in cholangiocarcinoma cells. Cells were treated with IC50 and 2*IC50 concentrations of silibinin for 48 h. Data is shown as mean ± SD, *p < 0.05, **p < 0.01, ***p < 0.001, significantly different compared to the control group. **(C)**. Mitochondrial membrane potential changes were observed by confocal microscopy after silibinin treatment. Red fluorescence indicates aggregates and green fluorescence indicates monomers. **(D)**. Colony formation assay results of HuCCT-1 and CCLP-1 cell lines treated with silibinin and SCH772984. **(E)**. EdU proliferation assay results of HuCCT-1 and CCLP-1 cell lines treated with silibinin and SCH772984.

From prior results, it can be determined that the apoptosis of cholangiocarcinoma cells is associated with mitochondria whose reduced membrane potential can cause cell apoptosis. Changes of the mitochondrial membrane potential is related to ERK protein expression. Therefore, the expression of ERK and p-ERK were initially examined. The expression of both proteins was significantly reduced after silibinin administration (Figures 4A,C). At the same time, the ratio of p-ERK to ERK also significantly decreased, which means that silibinin treatment was associated with ERK degradation and possibly ERK dephosphorylation (Supplementary Figure S1B). Additionally, Phosphorylated ERK can indirectly activate Bcl-2 and Bcl-xl protein on the downstream mitochondrial membrane, in order to change the mitochondrial membrane potential. As hypothesized, a decrease of p-ERK expression led to a significant decrease in the expression of Bcl-2 and Bcl-xl. The decrease of Bcl-2 and Bcl-xl protein led to a reduction in the mitochondrial membrane potential, as indicated by JC-1 assay, making JC-1 unable to aggregate within the matrix of mitochondria, leading to a transition from aggregates to monomers (Figures 5A,B). Consistent with FCM results, confocal microscopy results demonstrated a significant transition from red signal to green signal after silibinin treatment (Figure 5C). A reduction in mitochondrial membrane potential was a hallmark event within the early stages of apoptosis, which can further trigger release of Cytochrome C from mitochondria to cytoplasm. Cytochrome C was significantly decreased within the mitochondria, but increased in the cytoplasm after silibinin administration, which further activated downstream Caspase-9, causing cholangiocarcinoma cell apoptosis (Figures 4B,C).

### ERK Regulated Silibinin-Mediated Apoptosis in Cholangiocarcinoma Cells

**FIGURE 6:**
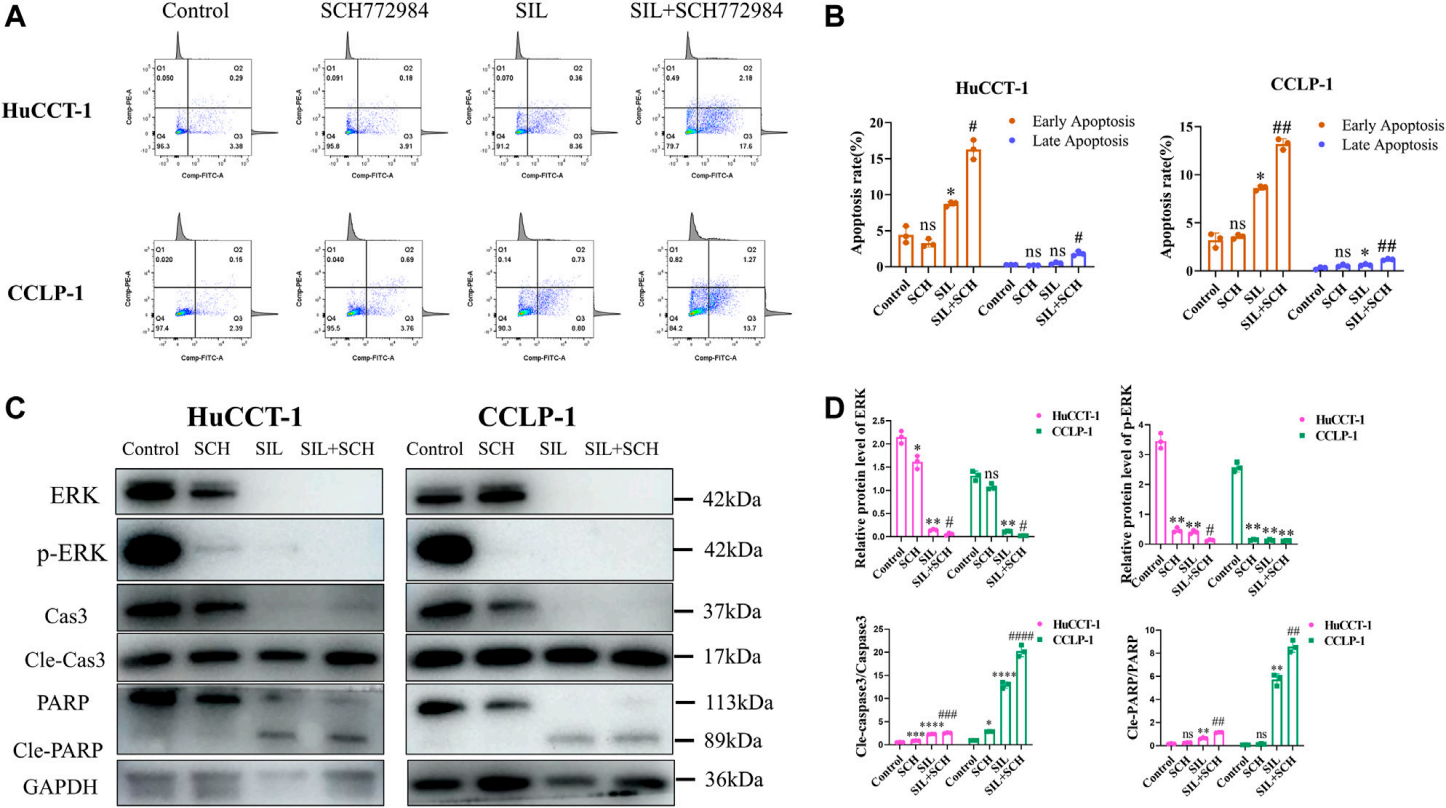
The combined application of SCH772984 significantly aggravated apoptosis of cholangiocarcinoma cells. **(A,B)**. Apoptosis detection by FCM and quantitative analysis of the apoptosis rate. **(C,D)**. The expression of apoptosis-related proteins was identified in HuCCT-1 and CCLP-1 cell lines, and quantification results were shown by histograms. Data is shown as mean ± SD, *p < 0.05, **p < 0.01, ***p < 0.001, ****p < 0.0001, significantly difference compared to the control group. #*p < 0.05*, ##*p < 0.01*, ###*p < 0.001,* significantly difference compared to the SIL group.

From these results, we speculated that ERK proteins have an important function in silibinin-mediated apoptosis of cholangiocarcinoma cells. Therefore, SCH772984, which is an inhibitor of ERK proteins, was utilized to explore the effect of selective inhibition of ERK on cholangiocarcinoma cells. The colony formation and proliferation ability of cholangiocarcinoma cells were significantly inhibited *via* silibinin, and the inhibitory effect was more significant after the addition of SCH772984 (Figures 5D,E). The cholangiocarcinoma cell apoptosis caused by silibinin was significantly aggravated following SCH772984 treatment (Figures 6A,B). In addition, the combination of silibinin and SCH772984 led to a significant increase in the expression of apoptosis-related proteins (Figures 6C,D).

### Silibinin Suppressed Growth of Tumors *In Vivo*

**FIGURE 7:**
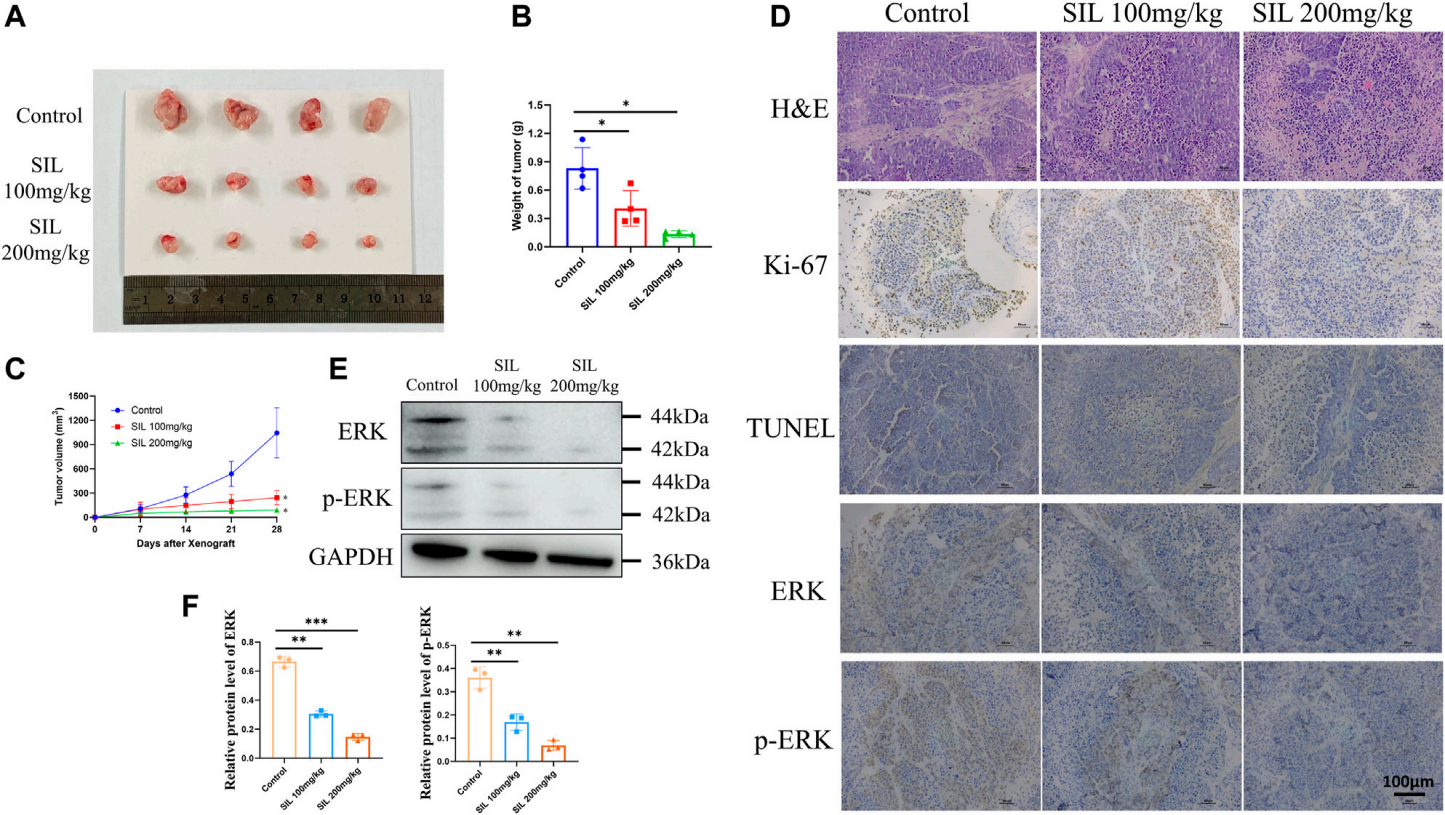
Silibinin suppressed tumor growth in the cholangiocarcinoma xenograft model. **(A)**. Anti-tumor activity of silibinin in tumor-bearing mice. Representative xenografts are shown. **(B)**. Tumor weights of each group measured at the end of treatment. Data are shown as mean ± SD, *p < 0.05, significantly different compared to the control group. **(C)**. Tumor volumes of each group are measured in the indicated days of treatment. *p < 0.05 meant a significant difference compared to the control group at the end of the treatment. **(D)**. H&E, Ki-67 and immunohistochemistry assays of cholangiocarcinoma tissue. **(E,F)**. The expression of ERK and p-ERK proteins was detected *in vivo,* and quantification results are shown by histograms. Data is shown as mean ± SD, **p < 0.01, ***p < 0.001, significantly different compared to the control group.

In order to investigate the effect of silibinin in cholangiocarcinoma growth *in vivo*, mice bearing cholangiocarcinoma subcutaneous xenografts were utilized. After silibinin was administered for 3 weeks, tumor size and weight were significantly reduced (Figures 7A,B). From the 14th day after silibinin treatment, the tumor growth rate was significantly reduced compared to the control group. Additionally, the greater the dose administered, the more significant the difference (Figure 7C). IHC analysis indicated a significant reduction of Ki-67 staining, as well as a dramatic increase of TUNEL staining in tumor tissues of mice treated with silibinin, suggesting that tumor proliferation was inhibited and apoptosis played an important role (Figure 7D, Supplementary Figure S1C). Finally, ERK and p-ERK protein expression in tumor tissues was detected. Similar to the results *in vitro*, silibinin was able to significantly reduce expression of two proteins (Figures 7D–F, Supplementary Figure S1C). These results demonstrate that silibinin suppressed cholangiocarcinoma growth *in vivo* in a similar manner.

## Discussion

Silymarin, a traditional Chinese medicine, is the dried and mature fruit of *Silybum marianum Gaertn*, with several functions including bring down a fever, detoxifying, soothing the liver and gallbladder (Federico et al., 2017). Silibinin, which is extracted from the shell of silybin seed, is one of the components with the highest content, as well as the strongest anti-inflammatory activity (Chen J et al., 2020). Due to its effect as an antioxidant (Chen YH et al., 2020), anti-lipid peroxidation, anti-fibrosis (Liu R et al., 2020) and immune regulation (Luo et al., 2019), silibinin is known to be a “natural liver protecting medicine”. It is widely used in the treatment of hepatitis, liver cirrhosis and metabolic liver injury (Abenavoli et al., 2010). In recent years, studies have demonstrated that silibinin has activities other than liver protection, including a strong anti-tumor activity, which has attracted more attention from researchers (Bosch-Barrera et al., 2017). Cholangiocarcinoma is a type of tumor with high molecular and genetic heterogeneity, as its location can occur anywhere from ductus arteriosus to the common bile duct (Rizvi et al., 2018). Due to the different molecular pathology and genetic mutations between tumors of the biliary system across different anatomic locations, it is more difficult to treat cholangiocarcinoma compared to other neoplasms (Rizvi and Gores, 2013). From the perspective of cell biology, the process of carcinogenesis involves cell proliferation, differentiation, apoptosis, angiogenesis and as well as others. From the perspective of molecular biology, it involves a series of proteins, kinases, transcription factors and cellular pathways. Silibinin can have an important function across different stages of carcinogenesis, including inhibiting proliferation of cancer cells, regulating cell cycle and inducing apoptosis in order to play a considerable role in anti-tumor activity (Bosch-Barrera and Menendez, 2015). Many studies have demonstrated that silibinin has promising anticancer effects across both androgen dependent and independent prostate, skin, bladder, lung, colon, breast and liver cancers (Polachi et al., 2016; Vue et al., 2016; Barros et al., 2020; Liu Y et al., 2020). However, thus far, there have been no reports on the application of silibinin on cholangiocarcinoma treatment. With this in mind, this study utilized silibinin in the treatment of cholangiocarcinoma for the first time and explored the underlying mechanisms in order to search for a new therapeutic approach for cholangiocarcinoma.

The cell cycle is a fundamental process of cellular life activities, fueled by the sequential activation of corresponding cyclin-dependent kinases (CDKs) by cyclins (Dalton, 2015). Cyclin periodic accumulation and disassembly positively regulates cell cycle progression, whereas CDK inhibitors negatively regulate cell cycle progression by inhibiting activity of CDKs at appropriate time points during the cell cycle. Cyclins, CDKs, and CDK inhibitors coordinate with each other in order to regulate cell cycle progression (Kar, 2016). Silibinin caused G1 arrest by decreasing the expression of Cyclin D1, Cyclin E2 and CDK4 and inducing increased expression of p21 and p15 in the human pancreatic adenocarcinoma cell line SW1990 (Zhang et al., 2018). In the colon cancer cell line HT-29, a significant dose-dependent G1 arrest was observed after treatment with silibinin at doses of 50–100 μg/ml. On the other hand, a G2 arrest occurred after a prolonged (48 h) exposure to high doses of silibinin (Agarwal et al., 2003). Similar to these findings, our results revealed a significant G1 arrest in cholangiocarcinoma cell line when treated with silibinin at IC50 and 2*IC50 concentrations for 48 h. This was different from prior reports that indicated that silibinin led to a G2 arrest in tumors, including cervical (You et al., 2020) and gastric cancer (Zhang et al., 2013). This illustrates that there are different mechanisms of action of silibinin across different tumors and that silibinin mainly acts as a tumor suppressor by causing G1 arrest in cholangiocarcinoma.

Autophagy occurs continuously at a basal rate under normal cellular conditions in order to maintain a balance of intracellular recycling and metabolic regulations (Mizushima and Komatsu, 2011). The normal level of autophagy in cells is low, which is needed to maintain normal physiological metabolism. However, when itis activated by external adverse factors, it may cause cell autophagic death (Kim and Lee, 2014). Cells need to undergo many changes related to metabolic machinery before they become cancerous, and autophagy becomes downregulated during carcinogenesis. Cancer cells are able to express a panel of anti-autophagy genes, such as *Bcl-2*, *Akt*, and *PI3KC1*, suggesting that autophagy can inhibit the transformation of normal cells into cancer cells. Most autophagy regulators are oncogenes and tumor suppressors, which is also a reason why autophagy in cancer has not been fully defined. In glioma cell lines U87 and U251, silibinin promoted the up-regulated expression of BNIP3, as well as translocation of the protein into the mitochondria by activating autophagy. This, in turn, led to the translocation of AIF from the mitochondria into the nucleus, and ultimately caused cell death (Wang et al., 2020). In breast cancer, silibinin was able to induce autophagy in MCF-7 cells by decreasing Akt/mTOR expression, thus enhancing its inhibitory effect on estrogen receptors and finally causing breast cancer cell death (Zheng et al., 2015). We observed the same phenomenon in cholangiocarcinoma after treatment with silibinin. There was significant dose-dependent autophagy induced by silibinin in cholangiocarcinoma (Data not shown). We suggested that there was a relationship between autophagy and apoptosis induced by silibinin, but we did not validate this conjecture, which is also a limitation in our experiments.

ERK is a member of the MAPK family, and an important related molecule downstream of EGFR (Corcoran et al., 2012). After phosphorylation on threonine and tyrosine residues of ERK, p-ERK (the active form of ERK) enters the nucleus where it phosphorylates several substrates for other kinases and transcription factors. It eventually causes cell cycle progression, as well as changes in cell proliferation, differentiation, metabolism, apoptosis and other behaviors. Unlike the high mutation rates of *RAS* (Prior et al., 2020), *BRAF* (Davies et al., 2002), and others genes in cancer cells, acquired mutations in *ERK* have thus far been virtually absent in cancer cells. The reason may be that ERK in cells is able to regulate many substrates (i.e., kinases and transcription factors), and once the drug resistance mutation occurs in *ERK*, the effective regulation of these substrates becomes lost, affecting normal cell activities and being unable to guarantee cell survival. Moreover, an increasing number of preclinical findings show that ERK inhibitors have better outcomes than RAF and MEK inhibitors (Hatzivassiliou et al., 2012; Qin et al., 2012). Recently, several ERK inhibitors have entered clinical studies, including GDC-0994, Ulixertinib (BVD-523), KO-947, LY3214996, and others (Blake et al., 2016; Sullivan et al., 2018). In the present study, we applied silibinin, a natural extract of silymarin, to effectively inhibit the expression of ERK, causing a reduction in the expression of anti-apoptotic proteins, such as Bcl-2 and Bcl-xl. The ratio of p-ERK to t-ERK density is a common indicator for the interpretation of ERK inhibition. From the ratio of p-ERK to total ERK, we found that there was no significant difference in the ratio between different groups. This phenomenon suggests that the decrease of p-ERK after silibinin treatment may be due to the decrease of t-ERK, rather than silibinin inhibiting the phosphorylation of ERK. The decreased protein expression led to a decrease in the mitochondrial membrane potential, which, in turn, triggered Cytochrome C efflux from the mitochondria and finally activated downstream Caspase-9, Caspase-3 and other apoptotic proteins, causing cell apoptosis.

Silibinin is poorly water-soluble, and nano-formulations of silibinin constructs have been reported to effectively improve the bioavailability of silibinin (Liu et al., 2017). Constructing nanoparticles with silibinin as the core in order to treat cholangiocarcinoma is also the next direction of our endeavors.

## Conclusion

For the first time, our study confirmed that silibinin can have an effective therapeutic effect on cholangiocarcinoma, such as treatment of other tumors, and its mechanism may be associated with intervening ERK and mitochondrial membrane potential. At the cellular level, silibinin treatment can significantly cause degradation and dephosphorylation of ERK protein, which further reduces cell membrane potential and causes outflow of Cytochrome C. The accumulation of Cytochrome C in the cytoplasm initiates the downstream apoptotic cascade, and causes cell death. This research inspired us to treat cholangiocarcinoma by intervening ERK in the future.

## Data Availability

The original contributions presented in the study are included in the article/Supplementary Materials, further inquiries can be directed to the corresponding authors.

## Supplementary Material

Supplementary Figure S1
**(A)**. Trypan blue staining results of the HuCCT-1 and CCLP-1 cell lines after mitochondrial isolation. **(B)**. The ratio of p-ERK to ERK related to Figure 4A. Data are shown as mean ± SD, * *p* < 0.05, ** *p* < 0.01, **** *p* < 0.0001, significantly difference compared to the control group. **(C)**. The quantification results of IHC in Figure 7D. Data are shown as mean ± SD, ** *p* < 0.01, *** *p* < 0.001, **** *p* < 0.0001, significantly different compared to the control group.

## Abbreviations

CCK-8, cell counting kit-8; CDKs, cyclin dependent kinases; EdU, 5-Ethynyl-2′- deoxyuridine; ERK, extracellular regulated protein kinases; FCM, flow cytometry; FITC, Fluorescein isothiocyanate isomer; HE, hematoxylin and eosin; IC50, half maximal inhibitory; IHC, immunohistochemistry; JC-1, 5,5',6,6'-Tetrachloro-1,1',3,3'-tetraethyl-imidacarbocyanine iodide; LC3B, microtubule-associated proteins light chain 3B; MAPK, mitogen activated protein kinase; PI, propidium iodine; PMSF, Phenylmethanesulfonyl fluoride; PVDF, polyvinylidene fluoride; TUNEL, TdT mediated dUTP Nick End Labeling; WB, western blotting.
