# Supplementary figures and images for: Silibinin Therapy Improves Cholangiocarcinoma Outcomes by Regulating ERK/Mitochondrial Pathway
Source: Front Pharmacol. 2022 Mar 23;13:847905. doi: 10.3389/fphar.2022.847905 (PMC8983842; doi:10.3389/fphar.2022.847905)

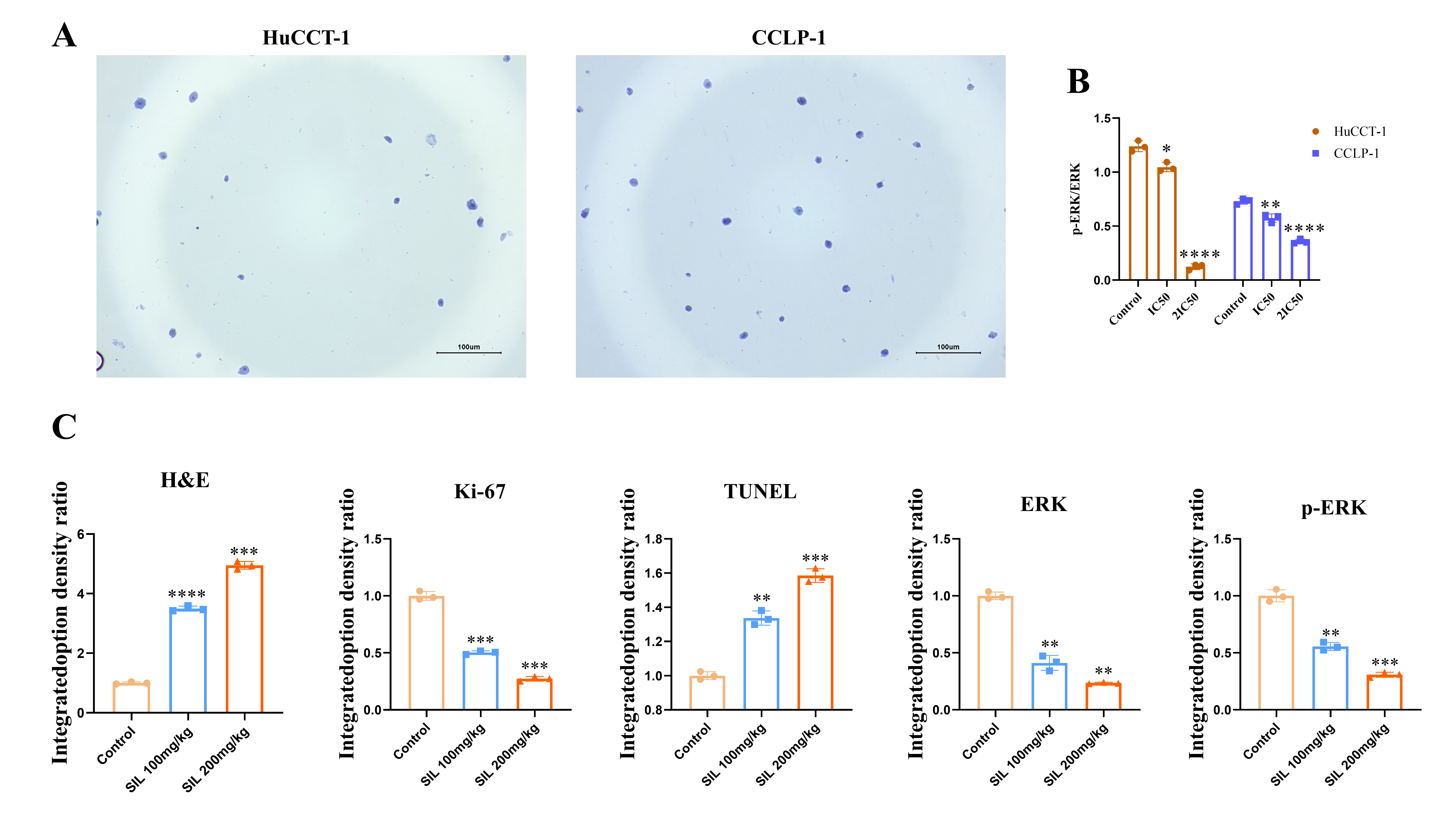

Supplement: Supplementary file 1 [file Image1.TIF]
